# Genetic Network Inference Using Hierarchical Structure

**DOI:** 10.3389/fphys.2016.00057

**Published:** 2016-02-23

**Authors:** Shuhei Kimura, Masato Tokuhisa, Mariko Okada-Hatakeyama

**Affiliations:** ^1^Department of Information and Electronics, Graduate School of Engineering, Tottori UniversityTottori, Japan; ^2^Laboratory for Integrated Cellular Systems, RIKEN Center for Integrative Medical SciencesYokohama, Japan

**Keywords:** genetic network, hierarchical random graph, hierarchical structure, bootstrap method, simulated annealing

## Abstract

Many methods for inferring genetic networks have been proposed, but the regulations they infer often include false-positives. Several researchers have attempted to reduce these erroneous regulations by proposing the use of a priori knowledge about the properties of genetic networks such as their sparseness, scale-free structure, and so on. This study focuses on another piece of a priori knowledge, namely, that biochemical networks exhibit hierarchical structures. Based on this idea, we propose an inference approach that uses the hierarchical structure in a target genetic network. To obtain a reasonable hierarchical structure, the first step of the proposed approach is to infer multiple genetic networks from the observed gene expression data. We take this step using an existing method that combines a genetic network inference method with a bootstrap method. The next step is to extract a hierarchical structure from the inferred networks that is consistent with most of the networks. Third, we use the hierarchical structure obtained to assign confidence values to all candidate regulations. Numerical experiments are also performed to demonstrate the effectiveness of using the hierarchical structure in the genetic network inference. The improvement accomplished by the use of the hierarchical structure is small. However, the hierarchical structure could be used to improve the performances of many existing inference methods.

## 1. Introduction

A genetic network is a functioning circuit in living cells at the gene level. From one viewpoint, a genetic network can be seen as an abstract mapping of an actual biochemical network consisting of genes, proteins, metabolites, and so on. The analysis of genetic networks is conceived as one of the promising ways to understand biological systems. The mathematical modeling of genetic networks has therefore become an important theme in systems biology.

Many studies have sought to develop computational methods for inferring genetic networks from observed gene expression patterns (Larrañaga et al., 2006; Chou and Voit, 2009; Hecker et al., 2009). Often, however, these methods infer false-positive regulations along with true-positive regulations. These erroneous regulations must be decreased if we are to successfully analyze the inferred genetic networks. One possible approach to remove these erroneous regulations from the inferred genetic networks is to use a priori knowledge about the networks. Several researchers have introduced a priori knowledge about the properties of genetic networks, such as their sparseness, scale-free structure, and so on, into methods for inferring genetic networks (see, e.g., Kikuchi et al., 2003; Daisuke and Horton, 2006).

This study focuses on another type of a priori knowledge, namely, that biochemical networks exhibit hierarchical structures (Clauset et al., 2008). The hierarchical structure in a network is a property having vertices that cluster together in groups, which then join to form groups of groups, and so forth, from the lowest levels of organization up to the level of the entire network. If we know the hierarchical structure in the target genetic network, we can improve a genetic network inferred by an inference method. That is, we can conclude that the regulations inferred by the method are unreasonable if they are inconsistent with the hierarchical structure. The hierarchical structure in a given network can be detected using a method based on the hierarchical random graph model (Clauset et al., 2008). While this detection method assumes that the erroneous regulations in a given network are infrequent, erroneous regulations actually tend to be abundant in a network inferred by a method for inferring genetic networks. Even if we simply used Clauset's method for the analysis of a genetic network, a reasonable hierarchical structure would be difficult to obtain.

In order to detect a hierarchical structure correctly, this study first infers multiple genetic networks from the observed gene expression data using a genetic network inference method in combination with a bootstrap method (Efron, 1979). We then extract a hierarchical structure from the inferred genetic networks that is consistent with most of the networks. As some erroneous regulations seem to be rarely inferred by the bootstrap method, we speculated that the proposed approach could reduce the effect of these erroneous regulations on the hierarchical structure detection. In this study, we extract a hierarchical structure from multiple genetic networks using the detection method proposed by Clauset et al. (2008) with modifications and then use the hierarchical structure obtained to assess the confidence values of the regulations. Through numerical experiments, we then demonstrate the effectiveness of the use of the hierarchical structure in the genetic network inference.

## 2. Detecting hierarchical structures

### 2.1. Hierarchical random graph model

Clauset et al. (2008) have proposed a method for detecting a hierarchical structure in a given network. Their method describes the given network as an undirected graph where the vertices and edges represent genes and interactions between them, respectively, in the genetic network inference. Note therefore that, while the method for inferring genetic networks generally treats a genetic network as a directed graph, the method for detecting hierarchical structures must treat it as an undirected graph.

**Figure 1:**
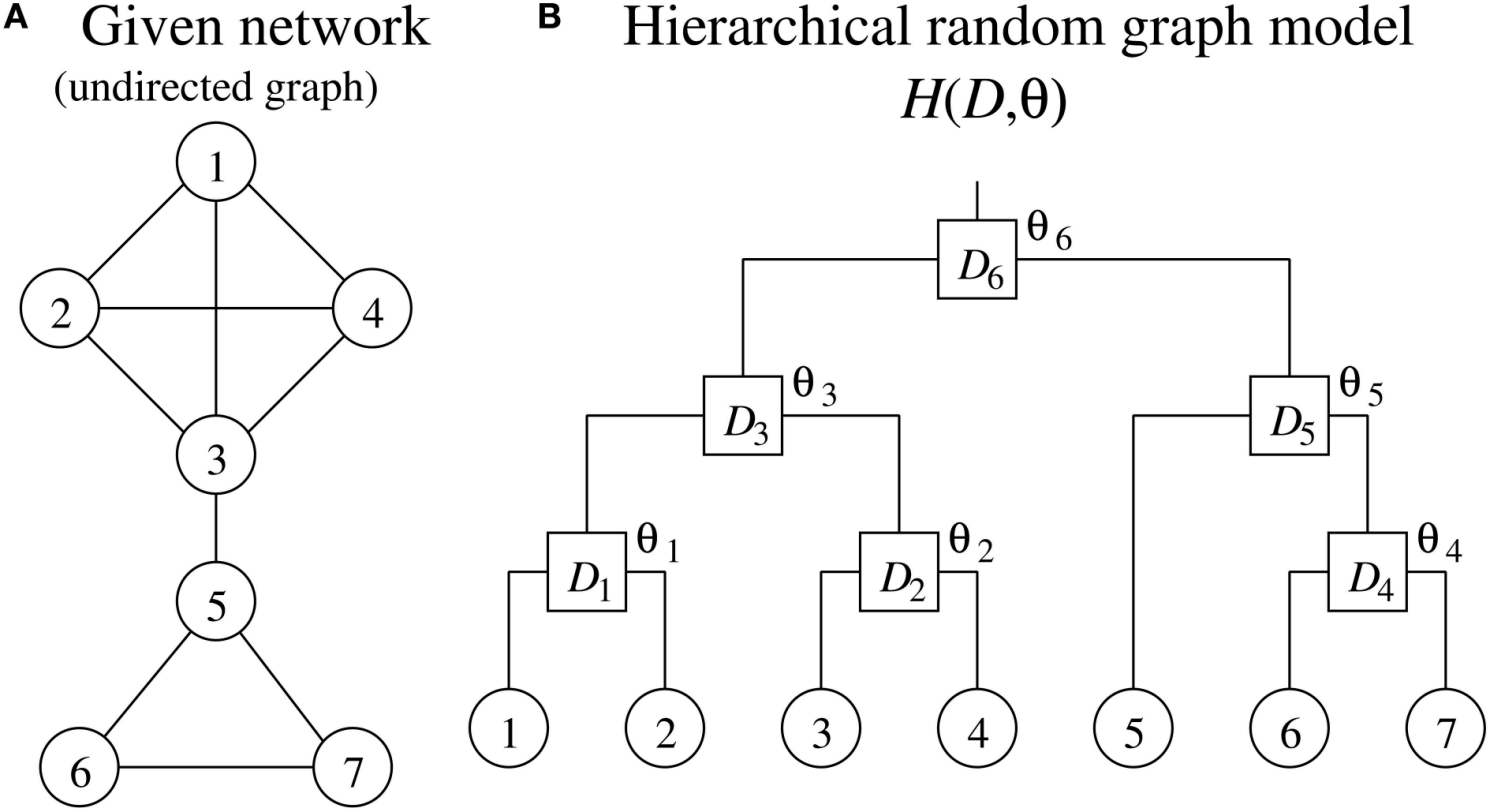
**(A)** A network and **(B)** the corresponding hierarchical random graph model.

The method proposed by Clauset et al. (2008) uses a hierarchical random graph model *H*(*D*, θ) to represent a hierarchical structure of a network consisting of *N* vertices, where *D* is a rooted binary tree having *N* leaf nodes and *N* − 1 internal nodes, and **θ** = (θ_1_, θ_2_, ⋯, θ_*N* − 1_) (see Figure 1). Each of the *N* leaf nodes of *D* corresponds to each of the vertices of the given network. The *N* − 1 internal nodes, which we represent here as *D*_1_, *D*_2_, ⋯, *D*_*N*−1_, indicate the hierarchical relationship among the vertices of the given network. Note that each pair of vertices in the given network has a unique internal node in *D* as their lowest common ancestor. The internal node *D*_*i*_ has a parameter θ_*i*_. The parameter θ_*i*_ represents the probability that the given network has an edge between vertices where *D*_*i*_ is the lowest common ancestor in *D*. When the vertices *u* and *v* have the internal node *D*_*i*_ as their lowest common ancestor, therefore, it means that the network has an edge between these vertices with the probability θ_*i*_. The model *H*(*D*, **θ**) has an ability to capture the hierarchical structure of the given network. On the other hand, *H*(*D*, **θ**) is also conceived as a generative model that allows us to generate artificial networks with a specified hierarchical structure.

The method proposed by Clauset et al. (2008) tries to find *D* and **θ** of *H*(*D*, **θ**), a model that serves well in representing the hierarchical structure of the given single network. The method proposed in this study, on the other hand, searches for them using multiple genetic networks inferred by the bootstrap approach.

### 2.2. Problem definition

The method proposed by Clauset et al. (2008) uses the maximum likelihood estimation for the hierarchical structure detection. Similarly, the method we propose here uses the maximum likelihood estimation to extract a hierarchical structure from the given networks. Here, therefore, we obtain the rooted binary tree *D* and the parameter vector **θ** by maximizing a probability that the given networks are generated from the model *H*(*D*, **θ**). The detection of the hierarchical structure in this study is thus defined as a maximization problem of the log-likelihood function (Kimura and Okada-Hatakeyama, 2015)

(1) $$\begin{array}{l} \text{log}L\left(D,\theta \right)=\overset{N_{g}}{\underset{j=1}{\sum }}\overset{N-1}{\underset{i=1}{\sum }}\left[E_{i}^{j}\text{log}\theta_{i}+\left(L_{i}R_{i}-E_{i}^{j}\right)\text{log}\left(1-\theta_{i}\right)\right], \end{array}$$

where *N*_*g*_ is the number of the given networks, *N* is the number of vertices contained in each network, and $E_{i}^{j}$ is the number of edges in the *j*-th network between vertices having *D*_*i*_ as the lowest common ancestor in *D*. *L*_*i*_ and *R*_*i*_ are the number of leaf nodes of the left and right subtrees, respectively, rooted at *D*_*i*_.

From the optimality conditions on the maximization problem of the function (Equation 1), i.e., $\frac{\partial \text{log}L}{\partial \theta_{i}}=0$, (*i* = 1, 2, ⋯, *N* − 1), we obtain

(2) $$\begin{array}{l} \theta_{i}=\frac{\sum_{j=1}^{N_{g}}E_{i}^{j}}{N_{g}L_{i}R_{i}},\left(i=1,2,\cdots ,N-1\right) \end{array}$$

The equations above indicate that the appropriate values for the parameters θ_*i*_'s are easily obtained for a given binary tree *D*. Our method thus extracts the hierarchical structure only by searching for the optimal *D*, as described below.

### 2.3. Optimization algorithm

The method proposed by Clauset et al. (2008) extracts a hierarchical structure from only a single network. The given data are insufficient, so many hierarchical random graph models seem to match the given network well. Their method thus generates multiple models using a Markov chain Monte Carlo method (Chib and Greenberg, 1995), and then averages them to obtain the hierarchical structure.

In our study, the use of multiple networks to detect the hierarchical structure allows us to search for a single optimum model using a simulated annealing (Kirkpatrick et al., 1983). Our method thus optimizes the objective function (Equation 1) according to the following procedure.

[Algorithm for maximizing the function (Equation 1)]

**Figure 2:**
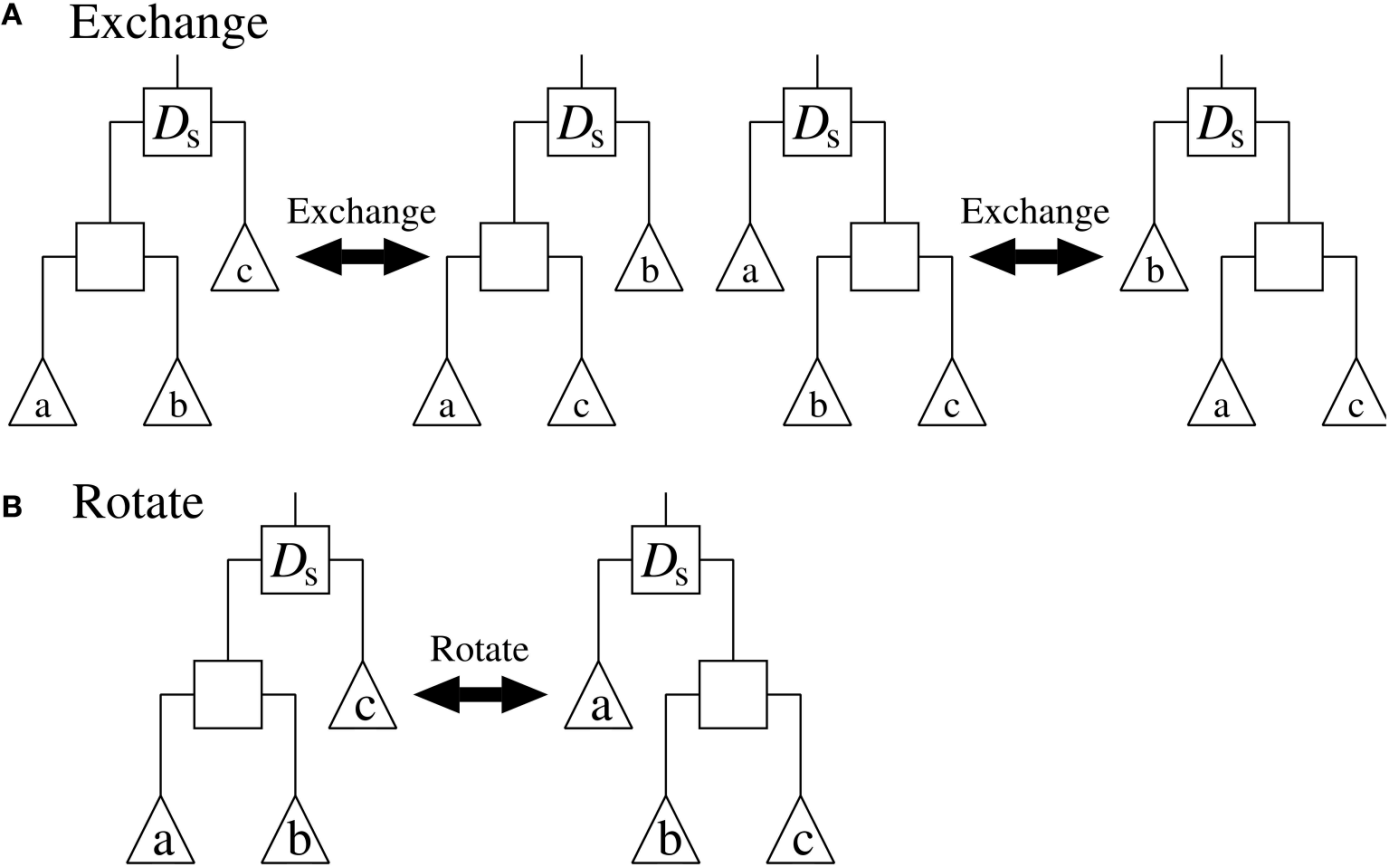
**The (A) “Exchange” and (B) “Rotate” operators applied to the internal node *D_**s**_***. Squares represent internal nodes, and triangles represent leaf nodes and/or subtrees.

1. Randomly generate a rooted binary tree, *BestTree*, with *N* leaf nodes, where *N* is the number of vertices contained in each of the given networks. Note that each leaf node of the tree corresponds to each vertex of the given networks. Compute the objective value of *BestTree* using the function (Equation 1). To compute this value for the function (Equation 1), we must first give the parameters θ_*i*_ along with a binary tree *D*. As mentioned in the section Problem Definition, however, this study directly computes the values for θ_*i*_ according to the Equation (2). Set *T* to *T_start_*.
2. Copy *BestTree* to *CurrentTree*.
3. Set *Counter* to 0.
4. Copy *CurrentTree* to *TestTree*.
5. Select an internal node of *TestTree* randomly. Then, modify the structure of *TestTree* by applying ‘Exchange’ or ‘Rotate’ randomly to the selected node. “Exchange” and “Rotate” are operators that alter the structure of the subtree rooted at the selected node, as shown in Figure 2. After the modification, compute the objective value of *TestTree*.
6. If the objective value of *TestTree* is better than that of *BestTree*, copy *TestTree* to *BestTree*.
7. Copy *TestTree* to *CurrentTree* with a probability
  $$\text{min}\left\{1,\;\exp\left(-\frac{Obj_{c}-Obj_{t}}{T}\right)\right\},$$
  where *Obj_c_* and *Obj_t_* are the objective values of *CurrentTree* and *TestTree*, respectively.
8. *Counter* ← *Counter* + 1.
9. Return to the step 4 if *Counter* < *N_max_*.
10. *T* ← γ*T*.
11. Return to the step 2 if *T* >*T_end_*. Otherwise, output *BestTree* and stop.

*T_start_*, *T_end_*, *N_max_*, and γ in the algorithm above are constant parameters. For this study, we set their values to 1000, 0.1, 1000*N*, and 0.99, respectively.

## 3. Assignment of confidence values to regulations

As mentioned previously, this study first infers *N_g_* genetic networks from the observed time-series of the gene expression levels. Any inference method capable of producing multiple genetic networks will serve this purpose. Here, however, we decided to use a method proposed by Kimura et al. (2010) for the generation of multiple genetic networks within a relatively short computation time by combining the LPM-based inference method (Kimura et al., 2009a) with the bootstrap method. We refer to this inference method as the BS-LPM inference method.

While the BS-LPM inference method distinguishes the regulation of the *n*-th gene from the *m*-th gene and vice versa, the method for detecting hierarchical structures described in the section Detecting Hierarchical Structures makes no such distinction. Here, therefore, we take the following step to transform the inferred genetic networks to the networks for our method for detecting the hierarchical structure: when the *j*-th genetic network inferred by the BS-LPM inference method contains the regulation of the *n*-th gene from the *m*-th gene, the regulation of the *m*-th gene from the *n*-th gene, or both, we add an edge between the *n*-th and *m*-th vertices to the *j*-th network for our detection method. The BS-LPM inference method is also capable of inferring an auto-regulation/auto-degradation, i.e., a regulation of a gene by itself. Here, however, we have to remove auto-regulations/auto-degradations from the networks, as our detection method cannot cope with them. Inferred networks usually contain auto-regulations/auto-degradations, because inference methods often infer the degradation of transcripts of a gene as a regulation of the gene by itself. We would not always need to search for regulations that usually exist. As such, the inference of auto-regulations/auto-degradations is not always essential for the inference of actual genetic networks. In order to detect a hierarchical structure in our target network, we next apply our detection method to the networks transformed above.

The confidence values of regulations can be evaluated solely based on the probabilities that the genetic networks inferred by the BS-LPM inference method contain the regulations. The hierarchical random graph model *H*(*D*, **θ**) in our method provides the probabilities that the target network has interactions between genes, which enable us to assign the confidence values to regulations on that basis, as well. We therefore try to improve the confidence values of regulations in this study by combining the probabilities evaluated by the BS-LPM inference method with those evaluated by *H*(*D*, **θ**). This study simply computes the combined confidence value of the regulation of the *n*-th gene from the *m*-th gene, *p_n, m_*, by

(3) $$\begin{array}{l} p_{n,m}= \eta p_{n,m}^{B}+ (1- \eta )p_{n,m}^{H}, \end{array}$$

where η (0 ≤ η ≤ 1) is a constant parameter, and $p_{n,m}^{B}$ and $p_{n,m}^{H}$ are the probabilities assigned to the regulation of the *n*-th gene from the *m*-th gene evaluated by the BS-LPM inference method and *H*(*D*, **θ**), respectively. Note here that *H*(*D*, **θ**) disregards the directions of regulations. While the values for $p_{n,m}^{B}$ and $p_{m,n}^{B}$ are basically different from each other, therefore, $p_{n,m}^{H}$ and $p_{m,n}^{H}$ always have the same value.

Note that the hierarchical random graph model *H*(*D*, **θ**) is extracted from the networks inferred by the BS-LPM inference method. Therefore, we should not depend too much on the results obtained from *H*(*D*, **θ**). In this study, thus, we mainly uses the extracted hierarchical structure to rank the regulations that are assigned the same probability value by the BS-LPM inference method. For this purpose, this study sets the parameter η to $1-\frac{1}{N_{g}}$.

## 4. Numerical experiments

### 4.1. Analysis of DREAM3 networks

From here, we will describe a series of experiments performed with five artificial genetic networks to check whether or not the use of the hierarchical structure is efficient for the inference of genetic networks.

#### 4.1.1. Experimental setup

**Figure 3:**
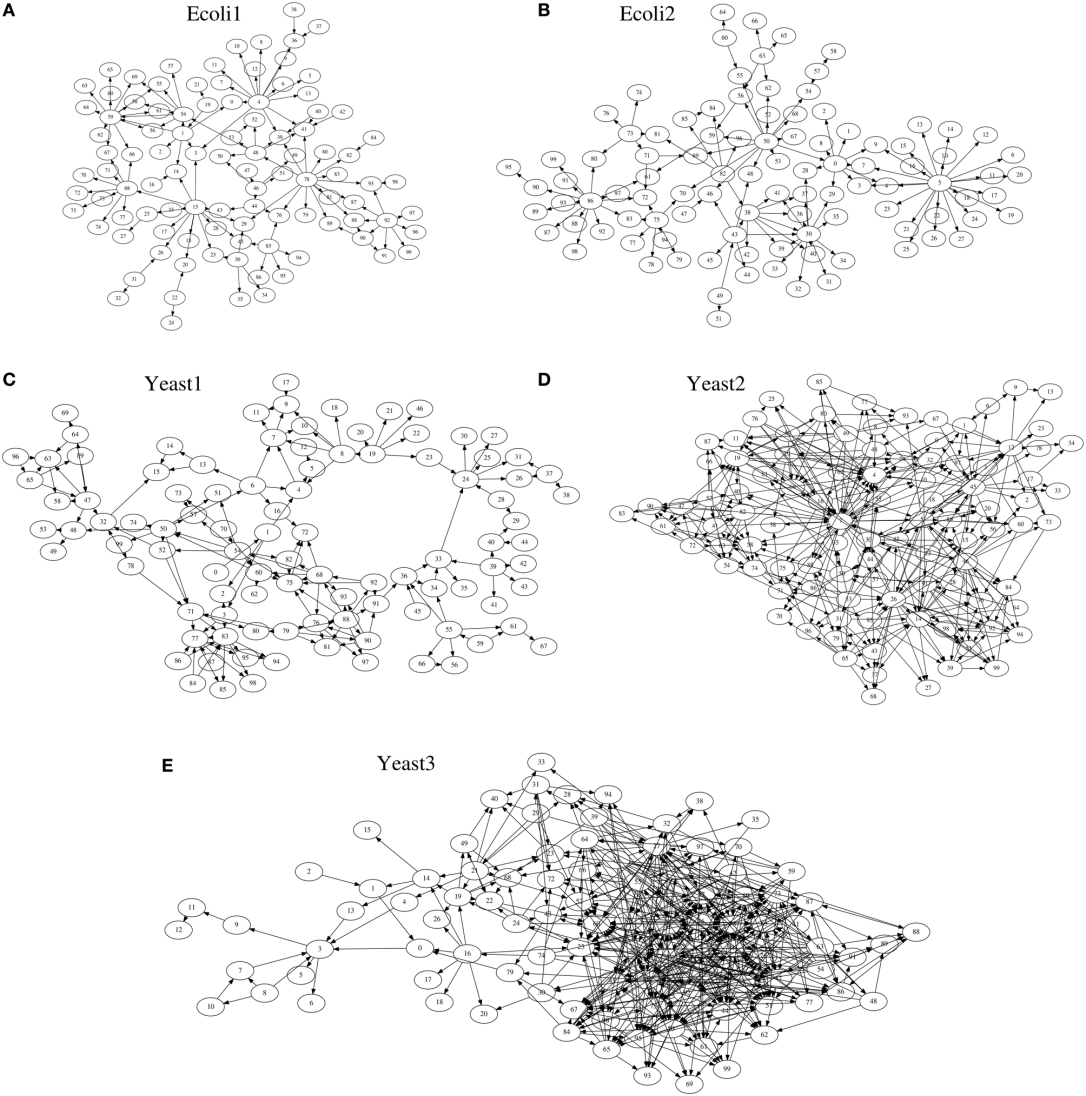
**The network structures of (A) Ecoli1, (B) Ecoli2, (C) Yeast1, (D) Yeast2, and (E) Yeast3**.

As target networks, we used a series of S-system models (Voit, 2000) consisting of 100 genes (*N* = 100), with topologies identical to those of the five networks provided by the DREAM3 *in silico* network challenges, i.e., Ecoli1, Ecoli2, Yeast1, Yeast2, and Yeast3 (http://dreamchallenges.org/) (Figure 3). The DREAM3 networks have often been used to check the performance of genetic network inference methods (see e.g., Lim et al., 2013). The design of these networks is based on actual biochemical networks and therefore reflects the actual topological properties. Note here that our method for detecting hierarchical structures only uses the topological properties of genetic networks inferred by a genetic network inference method. Although the target networks are artificial, the experiments we describe here could confirm the effectiveness of the use of the hierarchical structure for the genetic network inference. DREAM3, on the other hand, describes these networks using a model different from the S-system model (Prill et al., 2010). While the model used in DREAM3 considers the effect of the intrinsic noise, the S-system model disregards it. The BS-LPM inference method used in this study also disregards the intrinsic noise, so we used the S-system model to describe the target networks. Note that the purpose of the experiments here was not to assess the performance of the inference method but to check the effectiveness of the use of the hierarchical structure for the genetic network inference. We could therefore demonstrate the effectiveness of the use of the hierarchical structure even when using the S-system model.

The S-system model is a set of differential equations of the form

(4) $$\begin{array}{l} \frac{dX_{n}}{dt}=\alpha_{n}\overset{N}{\underset{m=1}{\prod }}X_{m}^{g_{n,m}}-\beta_{n}\overset{N}{\underset{m=1}{\prod }}X_{m}^{h_{n,m}}(n=1,2, \cdots ,N), \end{array}$$

where *X_n_* is the *n*-th state variable, *N* is the number of components in the network, and α_*n*_ (> 0), β_*n*_ (> 0), *g_n, m_*, and *h_n, m_* are model parameters. In the genetic network inference, *X_n_* is the expression level of the *n*-th gene and *N* is the number of genes contained in the target network. As the parameters *g_n, m_*'s and *h_n, m_*'s determine the topology of the network, we constructed the target networks by changing their values. In instances where the original DREAM3 network has the regulation of the *n*-th gene from the *m*-th gene, we chose a value for *g_n, m_* randomly from [−1, −0.5] ∪ [0.5, 1]. Otherwise, *g_n, m_* was set to 0.0. The parameter *h_n, n_* was set to 1.0 in order to simulate the auto-degradation, and the other *h_n, m_*'s (*n ≠ m*) were set to 0.0. The parameters α_*n*_ and β_*n*_ were all set to 1.0. We determined to use this parameter setting based on the reference (Kimura et al., 2009a). The numbers of regulations contained in Ecoli1, Ecoli2, Yeast1, Yeast2, and Yeast3, excluding auto-degradations, were 125, 119, 166, 389, and 551, respectively. As the inference ability of the proposed approach might depend on the values for the model parameters, we changed the random parameter values in every trial. Ten trials were performed on each of the five target networks.

As the observed gene expression patterns, 100 sets of time-series data, each covering 100 genes, were computed from the differential Equations (4) on each of the target models. The sets began from randomly generated initial values in [0.0, 2.0], and 11 observations with 0.4 time intervals between two adjacent observations were assigned to each gene in each set. In a practical application, these sets would be obtained by actual biological experiments under different experimental conditions. The measurement noise was simulated by adding 10% Gaussian noise to the computed time-series data. By applying the BS-LPM inference method (Kimura et al., 2010) to the generated gene expression data, we inferred 100 networks (*N_g_* = 100). We used the recommended values for the parameters of the BS-LPM inference method, namely, σ = 0.15, $C_{1}=\frac{200}{N\sqrt{K}}$, *C*_2_ = 0.4*C*_1_, and δ = 0.05, where *N* is the number of genes contained in the target network and *K* is the number of measurements. Thus, *N* = 100 and *K* = 100 × 11 = 1100 in these experiments.

In order to obtain a hierarchical random graph model *H*(*D*, **θ**), we then applied the hierarchical structure detection method described in the section Detecting Hierarchical Structures to the *N_g_* generated genetic networks. We then used the hierarchical random graph model obtained to compute the confidence values of the regulations, as described in the section Assignment of Confidence Values to Regulations. The constant parameter for computing the confidence values, η, was set to $1-\frac{1}{N_{g}}=0.99$. As mentioned previously, we mainly uses the extracted hierarchical structure to rank the regulations that are assigned the same confidence value by the BS-LPM inference method. This study therefore did not depend too much on the hierarchical structure *H*(*D*, **θ**).

#### 4.1.2. Results

As described previously, the proposed approach and the BS-LPM inference method were both capable of assigning the confidence values to all of the candidate regulations. In this study, we checked the performance of these methods by constructing a network of regulations whose confidence values exceeded a threshold and then comparing it with the target network. We checked the performance using the recall and the precision. The recall and the precision are defined as

$$\text{recall}=\frac{TP}{TP+FN},\text{ precision}=\frac{TP}{TP+FP},$$

where TP, FP, and FN are the numbers of true-positive, false-positive, and false-negative regulations, respectively. Note that we transformed the genetic networks inferred by the BS-LPM inference method into undirected graphs for detecting their hierarchical structure. When evaluating the performance, however, we distinguished the regulation of the *n*-th gene from the *m*-th gene and vice versa, i.e., we treated the networks as directed graphs. We also disregarded auto-regulations/auto-degradations in the evaluation.

**Figure 4:**
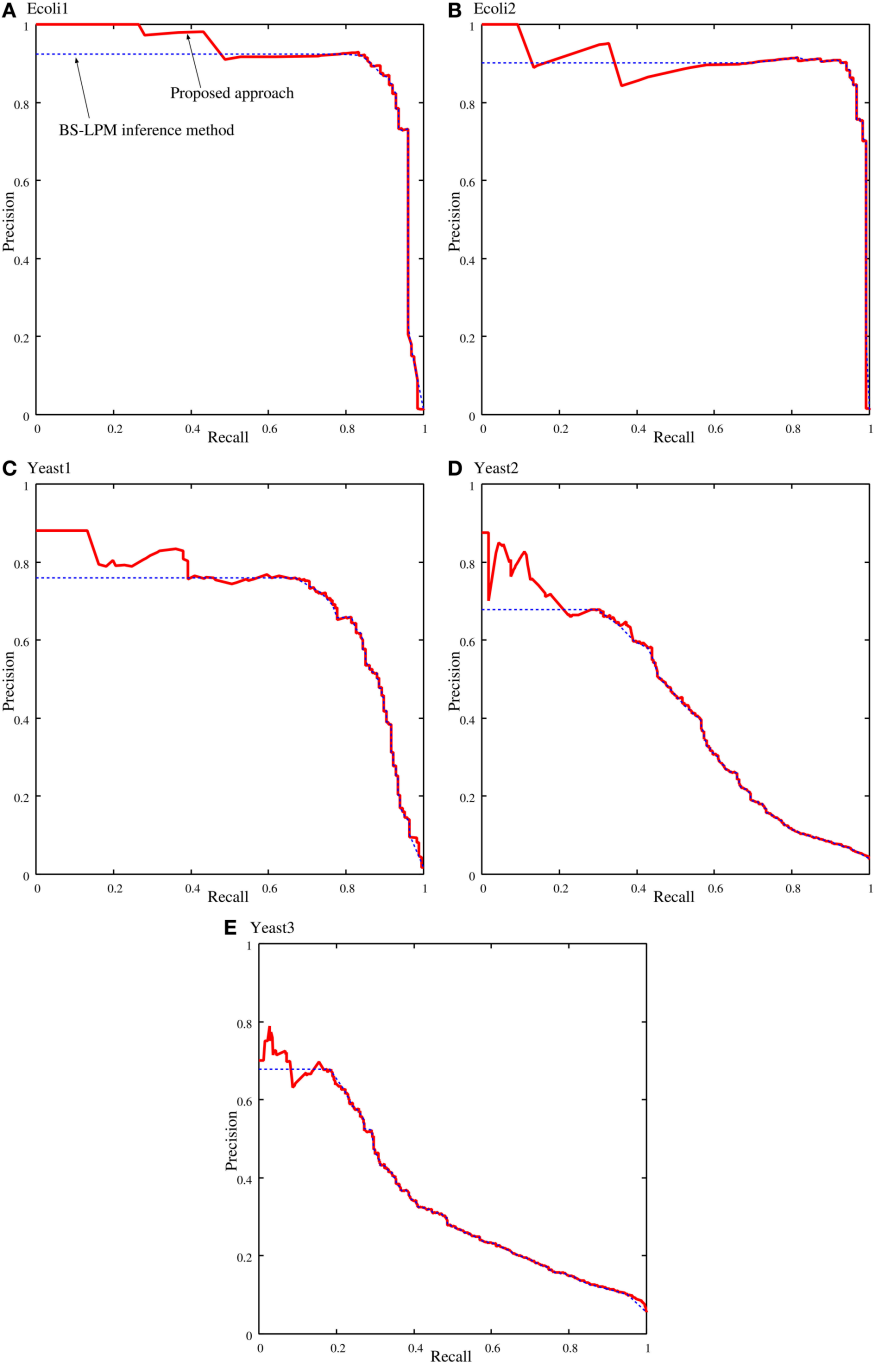
**Samples of the recall-precision curves of the proposed approach and the BS-LPM inference method on the problems of (A) Ecoli1, (B) Ecoli2, (C) Yeast1, (D) Yeast2, and (E) Yeast3**. Solid and dotted lines represent the performance of the proposed approach and the BS-LPM inference method, respectively.

Figure 4 shows samples of the recall-precision curves obtained by the proposed approach and by the BS-LPM inference method by changing the threshold for the confidence value. We previously described how closely our method depends on the BS-LPM inference method. As the figure shows, the performance of our approach was therefore similar to that of the BS-LPM inference method. Meanwhile, the figure also shows that the use of the hierarchical structure improved the precision of our approach. This higher precision is a preferable feature, since biologists must experimentally validate the inferred regulations in actual applications. The BS-LPM inference method required about 4.12 h on a personal computer (Core i5-4670) to obtain *N_g_* (=100) genetic networks from the given gene expression patterns. The hierarchical structure detection method described in the section Detecting Hierarchical Structures required about 2.91 h on the same computer to extract a hierarchical structure from the generated genetic networks.

We quantified the performance of the proposed approach and the BS-LPM inference method in this study using the area under the recall-precision curve (AURPC). Table 1 lists the averaged AURPCs of the two methods on the problems of Ecoli1, Ecoli2, Yeast1, Yeast2, and Yeast3. Our approach outperformed the BS-LPM inference method on most of the 5 × 10 = 50 trials with respect to the AURPC, but its performance was still inferior in seven of the trials. The inferior performance in those seven failed trials was presumably due to a failure of our approach to detect the hierarchical structures in the target networks. Four of the failed trials were performed on the Ecoli2 problem and the other three were performed on Yeast1, Yeast2, and Yeast3. As shown in Figure 3B, a number of genes in Ecoli2 are regulated by only single genes. Our approach failed to adequately analyze networks with this property, as some regulations erroneously inferred by the BS-LPM inference method easily caused the formation of erroneous gene clusters. Ecoli2 would model a network in which some transcriptional factors regulate most of the other genes. Note here that genes regulated by the same transcriptional factor often show expression patterns similar to each other. Inference methods generally perform poorly in discriminating genes of this type. One solution for this problem is to use some clustering technique to identify genes with similar expression patterns, group them together, and then infer the regulations between the clusters (see e.g., Kimura et al., 2005). There would thus be no need, in practical application, to detect hierarchical structures in networks with topological properties similar to Ecoli2.

**Table 1:** **The performance of the proposed approach and the BS-LPM inference method evaluated with respect to the area under the recall-precision curve (AURPC)**.

	**Ecoli1 AVG ± STD**	**Ecoli2 AVG ± STD**	**Yeast1 AVG ± STD**	**Yeast2 AVG ± STD**	**Yeast3 AVG ± STD**
Proposed approach	0.9042 ± 0.0402	0.9233 ± 0.0252	0.7140 ± 0.0515	0.4330 ± 0.0251	0.3563 ± 0.0386
BS-LPM inference method	0.8792 ± 0.0528	0.9154 ± 0.0207	0.6880 ± 0.0491	0.4151 ± 0.0207	0.3504 ± 0.0368

*AVG and STD represent the averaged value of the AURPCs and its standard deviation, respectively*.

As described in the section Assignment of Confidence Values to Regulations, this study uses the parameter η to combine the results from the BS-LPM inference method and those from the hierarchical random graph model. Therefore, we then checked the effect of the parameter η on the performance of the proposed approach. Table 2 shows the AURPCs of our approach with different values for η. The experimental results indicate that, although the use of the hierarchical structure has an ability to improve the confidence values of regulations, we should not rely too much on it.

**Table 2:** **The AURPCs of the proposed approach with different values for the parameter η**.

**Parameter** **η**	**Ecoli1** **AVG ± STD**	**Ecoli2** **AVG ± STD**	**Yeast1** **AVG ± STD**	**Yeast2** **AVG ± STD**	**Yeast3** **AVG ± STD**
1.000	0.8792 ± 0.0528	0.9154 ± 0.0207	0.6880 ± 0.0491	0.4151 ± 0.0207	0.3504 ± 0.0368
0.999	0.9043 ± 0.0402	0.9233 ± 0.0252	0.7140 ± 0.0515	0.4330 ± 0.0251	0.3563 ± 0.0386
0.995	0.9043 ± 0.0402	0.9233 ± 0.0252	0.7140 ± 0.0515	0.4330 ± 0.0251	0.3563 ± 0.0386
0.990	0.9042 ± 0.0402	0.9233 ± 0.0252	0.7140 ± 0.0515	0.4330 ± 0.0251	0.3563 ± 0.0386
0.950	0.8969 ± 0.0415	0.9159 ± 0.0272	0.6962 ± 0.0486	0.4269 ± 0.0249	0.3487 ± 0.0390
0.900	0.8898 ± 0.0430	0.9110 ± 0.0259	0.6832 ± 0.0459	0.4180 ± 0.0243	0.3412 ± 0.0390

*Note that the proposed approach with η = 1.000 is equivalent to the BS-LPM inference method and the parameter $\eta =1-\frac{1}{N_{g}}=0.990$ is our recommended setting*.

**Figure 5:**
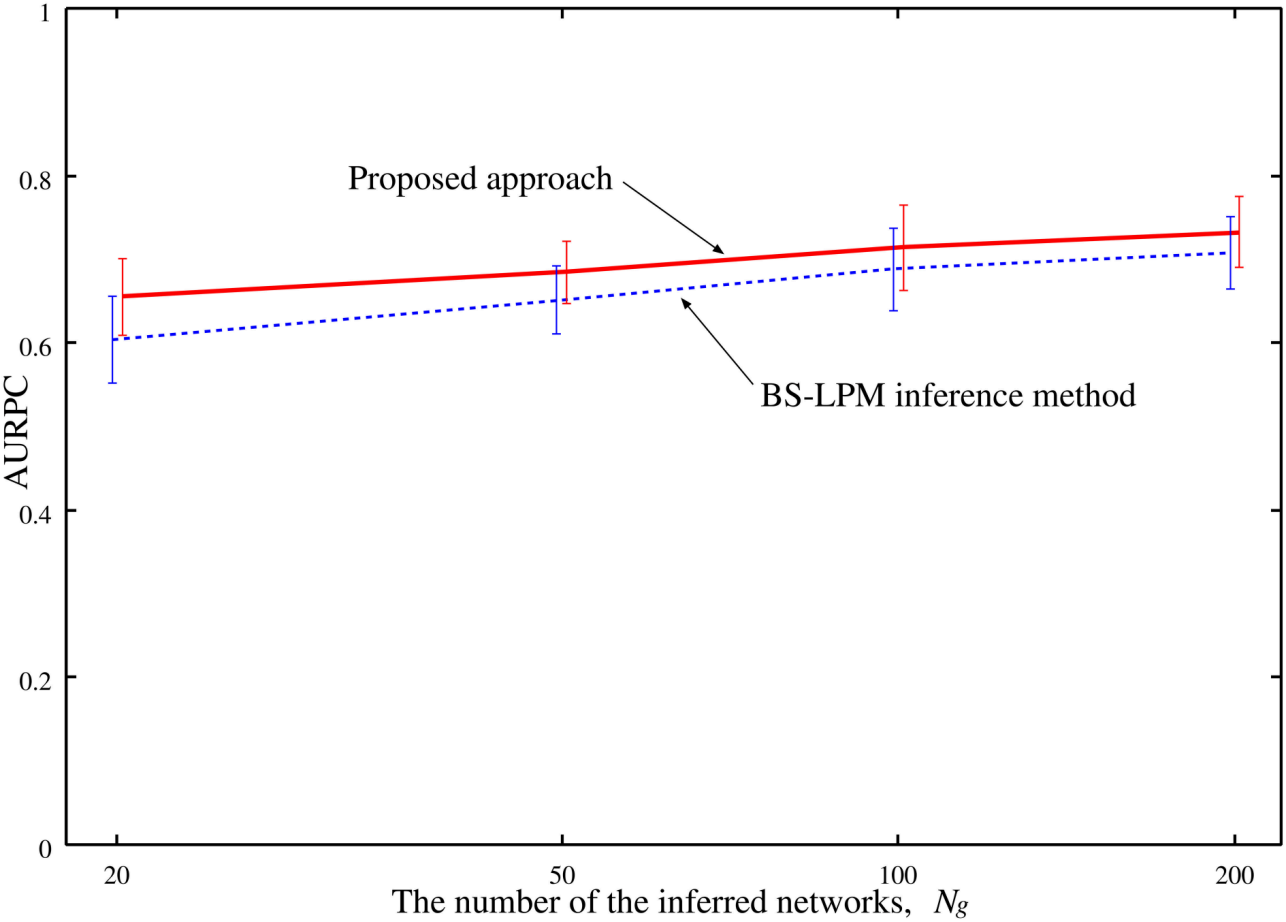
**The averaged AURPCs of the proposed approach (solid line) and the BS-LPM inference method (dotted line) plotted against the number of the inferred networks, *N_**g**_***.

The performance of the proposed approach might depend on the number of the networks inferred by the BS-LPM inference method, *N_g_*. Therefore, we also checked our setting of the parameter η, i.e., $\eta =1-\frac{1}{N_{g}}$, on the experiments with different numbers of *N_g_*. Figure 5 shows the AURPCs of the proposed approach with *N_g_* = 20, 50, 100, and 200 on the problems of Yeast1. The figure indicates the reasonableness of our parameter setting.

**Figure 6:**
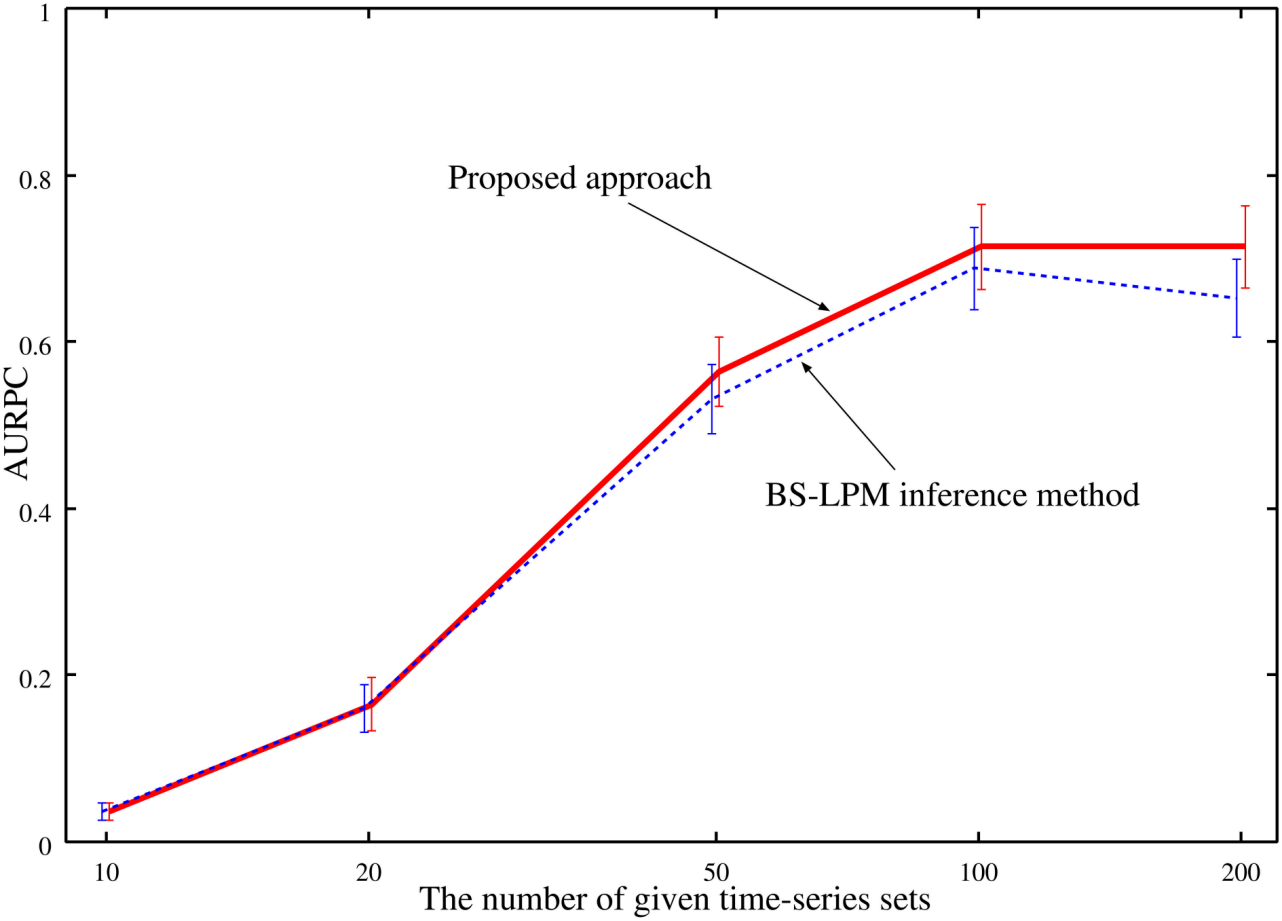
**The averaged AURPCs of the proposed approach (solid line) and the BS-LPM inference method (dotted line) in the experiments where different numbers of time-series sets were given**.

As mentioned previously, our approach improves the confidence values of regulations by combining the probabilities evaluated by the BS-LPM inference method with those evaluated by the hierarchical random graph model. Note that this study obtains the hierarchical random graph model using the genetic networks inferred by the BS-LPM inference method. Therefore, the reasonableness of the extracted hierarchical structure depends on the accuracy of the inferred genetic networks. We investigated how the accuracy of the inferred genetic networks affected the performance of the proposed approach by performing experimental runs with variable amounts of time-series data applied to the problems of Yeast1. Figure 6 plots the averaged AURPC against the amount of time-series data. The plot shows that the use of the hierarchical structure has no negative effect on the inference ability, on average, even when the inferred networks are inaccurate.

### 4.2. Analysis of an actual network

We next applied the proposed approach to an experiment using actual data.

#### 4.2.1. Experimental setup

This experiment analyzed an ErbB-receptor-mediated regulatory network of transcription factors in normal human epidermal keratinocytes. The network consisted of 29 components, i.e., three receptors (EGFR, ErbB2, and ErbB3), seven signal transducer proteins (ERK, PI3K, AKT, STAT3, PLCg, PKCd, and c-SRC), the phosphorylated forms of the three receptors and the seven signal transducer proteins, and seven transcription factors (c-FOS, FRA1, FRA2, JUNB, c-JUN, JUND, and c-MYC). Time-series data consisting of 14 measurements of the 29 components were measured by Saeki et al. (2012). Lacking sufficient data, we inferred the target network using the following a priori knowledge: (i) none of the receptors or signaling proteins are affected by other receptors or signaling proteins; (ii) none of the transcription factors are affected by receptors, signaling proteins, or phosphorylated forms of receptors; (iii) none of the phosphorylated receptors or phosphorylated signaling proteins are affected by other receptors, signaling proteins, or transcription factors; (iv) every component of this system regulates itself; (v) every protein regulates its own phosphorylated form. We employed this knowledge according to the biological knowledge that phosphorylated forms of signaling proteins and receptors can form cascades to transduce extracellular signals to transcription factors (Alberts et al., 2008). Based on the knowledge (i), for example, we prohibited inferring the regulation of EGFR from ErbB2. We used the technique proposed by Kimura et al. (2009b) in order to introduce the knowledge described above into the inference method. By introducing this a priori knowledge, we reduced the degree-of-freedom of the network model. The other experimental conditions were the same as those in the section Analysis of Dream3 Networks.

#### 4.2.2. Results

**Figure 7:**
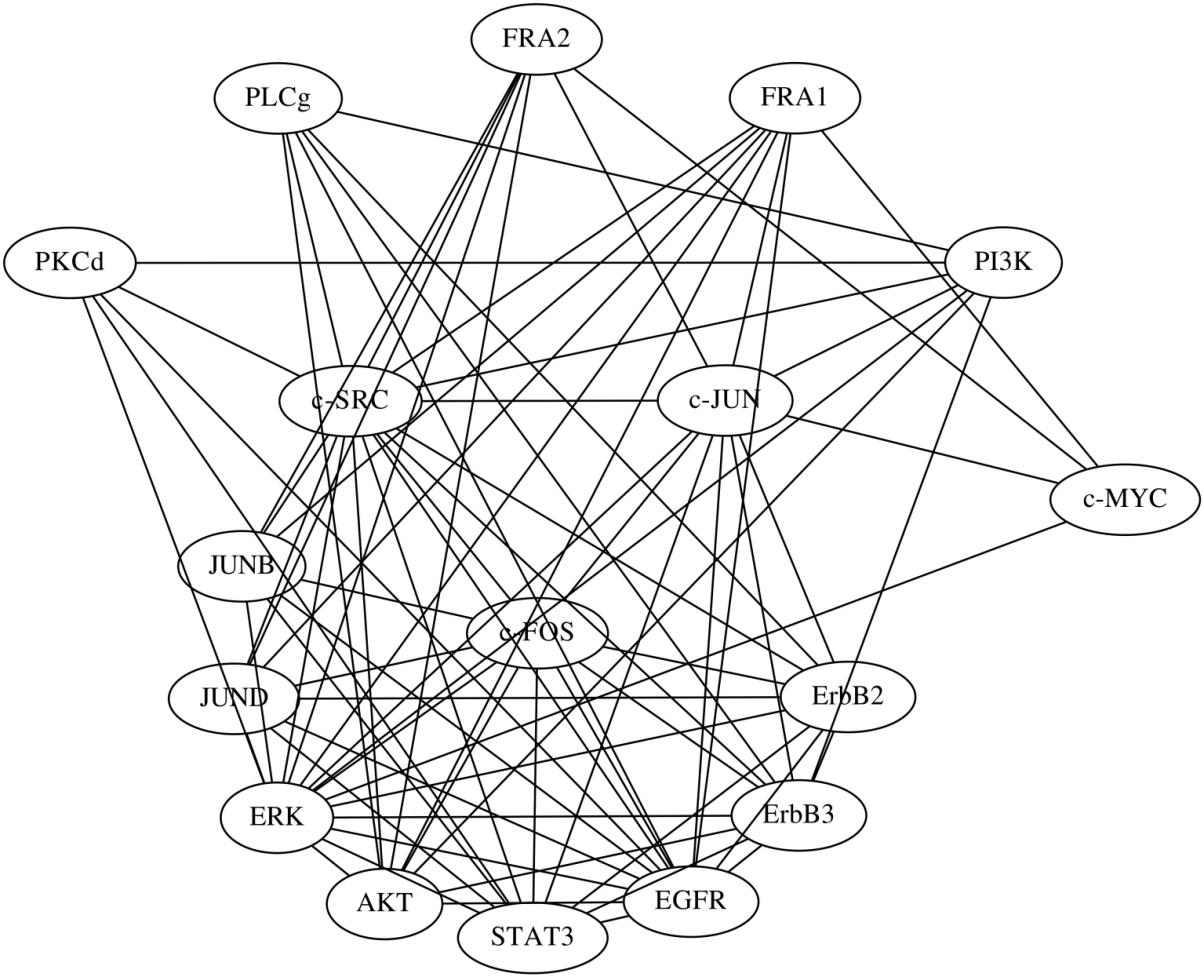
**The protein network obtained from the STRING database**. Edges represent protein-protein interactions that have been reportedly confirmed by biochemical experiments.

**Figure 8:**
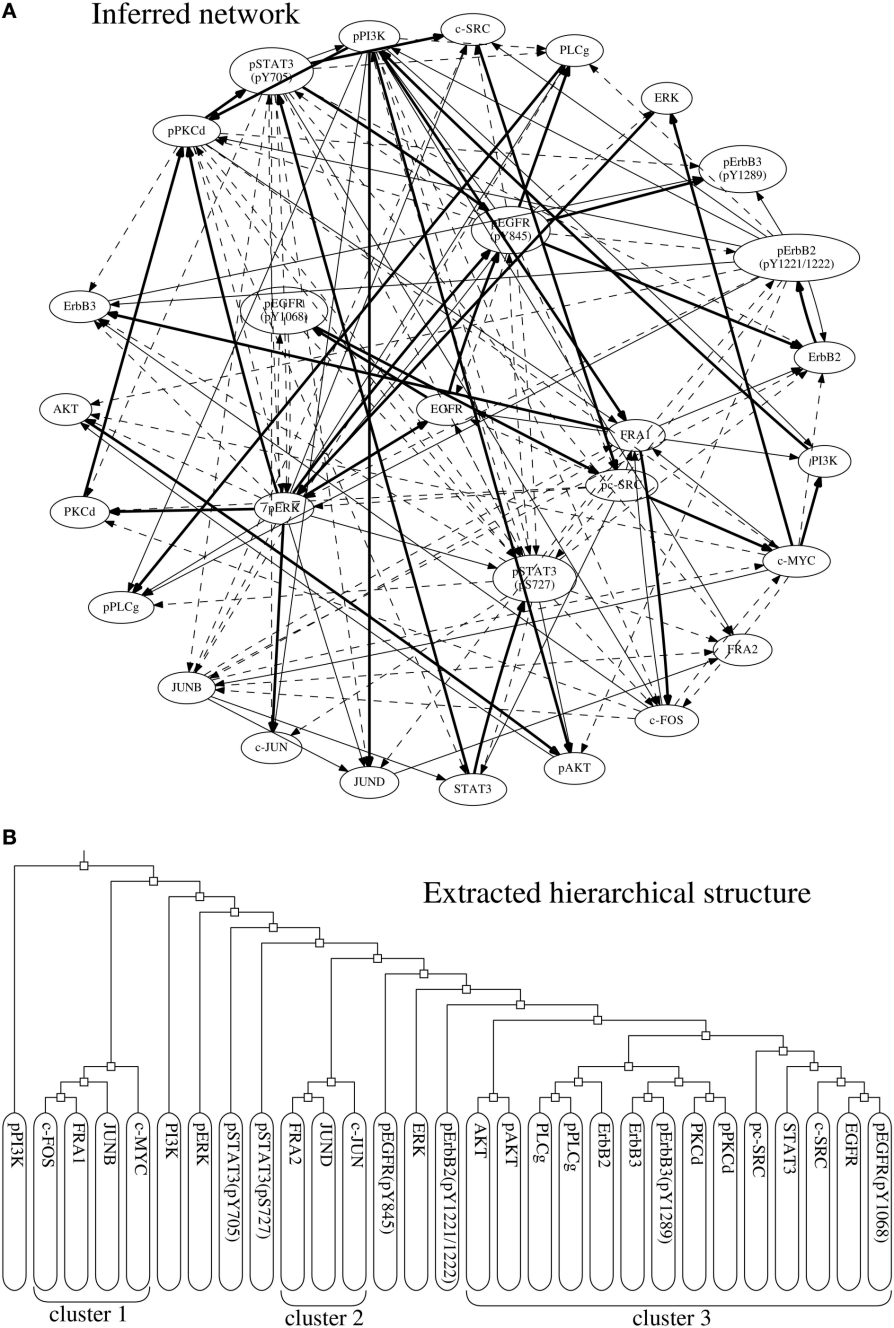
**(A)** The network of regulations with confidence values exceeding 0.25. Bold, solid, and dotted lines represent regulations with confidence values exceeding 0.75, 0.5, and 0.25, respectively. **(B)** The hierarchical structure extracted from the inferred networks.

The network of the regulations with confidence values exceeding 0.25 is shown in **Figure 8A**. The network obtained contained 135 regulations, but 17 were regulations of the proteins from their phosphorylated forms or vice versa, which probably made them trivial. We still lack a detailed understanding of the regulatory network used for this study, which consisted of proteins and their phosphorylated forms. We therefore compared the inferred network with a protein network consisting of the proteins alone (Figure 7). We obtained this protein network from the STRING database (http://string-db.org/) (Szklarczyk et al., 2015). The comparison results indicate that 77 of the 135 inferred regulations were reasonable, since the interactions between the corresponding proteins have been reportedly confirmed.

The proposed approach extracted a hierarchical structure of the target network from the networks inferred by the BS-LPM inference method. The extracted hierarchical structure is shown in Figure 8B. As the figure indicates, the network contained three clusters, i.e., clusters 1, 2, and 3. Clusters 1 and 2 contained the transcription factors, the downstream components of the target pathway. Cluster 3 mainly contained the upstream components. The phosphorylated ERK and the phosphorylated STAT3's, none of which belonged to any cluster, were intermediate components thought to regulate the transcription factors (Saeki et al., 2012). Although imperfect, the hierarchical structure obtained seemed to reflect the actual structure of the target pathway. We thus think that the hierarchical random graph model obtained can be used to assess the reliability of the inferred network and/or to understand the structure of the target network.

As mentioned before, our approach is highly dependent on the BS-LPM inference method. The inferred network was therefore almost the same as that obtained only from the BS-LPM inference method. Our approach improves the confidence values of the regulations using the hierarchical random graph model obtained. We know, for example, that the phosphorylated ERK and phosphorylated STAT3 regulate each other (Gao and Horvath, 2008). In this experiment, these regulations were inferred by both the proposed approach and the BS-LPM inference method. The BS-LPM inference method assigned a confidence value of 0.29 to the regulation of the phosphorylated STAT3 by the phosphorylated ERK, and assigned the same value to four other regulations. Our approach, on the other hand, assigned a confidence value of 0.2922 to the regulation of the phosphorylated STAT3 by the phosphorylated ERK, a value superior the confidence values assigned to the same four other regulations. This feature of our approach could be useful for reducing the efforts of biologists to experimentally validate inferred regulations.

## 5. Conclusion

In this paper, we have proposed an approach for inferring a more reasonable genetic network by utilizing the hierarchical structures in genetic networks. The first step of this new approach is to infer multiple genetic networks from the given gene expression data. In this study, we took this step using the BS-LPM inference method (Kimura et al., 2010). The next steps in our approach are to extract the hierarchical structure in the target network from the genetic networks generated in the first step, and then to use the extracted hierarchical structure to compute the confidence values of the regulations. Our experimental results showed that the use of the hierarchical structure improves the confidence values of the regulations. As mentioned in the section Assignment of Confidence Values to Regulations, however, this study used the obtained hierarchical structure to rank the regulations that are assigned the same probability by the BS-LPM inference method. When there are no regulations that have the same bootstrap probability, therefore, the use of the hierarchical structure does not work. In our future work, thus, we must improve this drawback.

The approach proposed in this study consists of a BS-LPM inference method and a method for detecting hierarchical structures. The BS-LPM inference method is a combination of the LPM-based inference method (Kimura et al., 2009a) and the bootstrap method. We have the freedom, however, to use any inference method in place of the LPM-based inference method. Meanwhile, several investigators have proposed other inference methods that are capable of assigning confidence values to regulations without the use of the bootstrap method (see e.g., Huynh-Thu et al., 2010). The use of the hierarchical structure may also be effective in improving the performance of these methods.

Several inference methods that utilize a priori knowledge about the properties of genetic networks have been already proposed (see e.g., Kikuchi et al., 2003; Daisuke and Horton, 2006). These methods use the a priori knowledge during the genetic network inference. We could say, on the other hand, that the proposed approach uses the a priori knowledge after inferring genetic networks. Our experimental results proved that, even after the genetic network inference, the use of the a priori knowledge has an ability to improve the confidence values of regulations. Thus, although the improvement done by the proposed approach was very small, our framework might enable us to use other types of a priori knowledge that are currently difficult to utilize.

## Author contributions

SK designed the method and performed the experiments. MT implemented some parts of the proposed algorithm. MOH supervised the biological aspect of this work. All authors read and approved the manuscript.

### Conflict of interest statement

The authors declare that the research was conducted in the absence of any commercial or financial relationships that could be construed as a potential conflict of interest.
